# LncRNA SCAMP1 disrupts the balance between miR-26a-5p and ZEB2 to promote osteosarcoma cell viability and invasion

**DOI:** 10.3389/fonc.2022.967000

**Published:** 2022-08-03

**Authors:** Rong Li, Zhen Chen, Yubo Zhou, Gulikezi Maimaitirexiati, Qi Yan, Yuting Li, Adilijiang Maimaitiyimin, Changhui Zhou, Jingqin Ren, Chengqing Liu, Abasi Mainike, Peng Zhou, Lu Ding

**Affiliations:** ^1^ College of Public Health, Xinjiang Medical University, Urumqi, China; ^2^ Department of Orthopedics, Traditional Chinese Medicine Hospital Affiliated to Xinjiang Medical University, Urumqi, China; ^3^ CAS Key Laboratory of Quantitative Engineering Biology, Shenzhen Institute of Synthetic Biology, Shenzhen Institutes of Advanced Technology, Chinese Academy of Sciences, Shenzhen, China; ^4^ Department of Orthopedics, Xinjiang Medical University Affiliated Fifth Hospital, Urumqi, China; ^5^ Traditional Chinese Medicine Hospital Affiliated to Xinjiang Medical University, Postdoctoral Research Center on Public Health and Preventive Medicine, Xinjiang Medical University, Xinjiang, China

**Keywords:** miR-26a-5p, SCAMP1, ZEB2, viability, invasion

## Abstract

Osteosarcoma often occurs in children and adolescents and affects their health. The survival rate of osteosarcoma patients is unsatisfactory due to the lack of early detection and metastasis development and drug resistance. Hence, dissection of molecular insight into osteosarcoma initiation and progression is pivotal to provide the new therapeutic strategy. In recent years, long noncoding RNAs (lncRNAs) have burst into stage in osteosarcoma development and malignant behaviors. LncRNA SCAMP1 has been discovered to play an essential role in carcinogenesis and progression. However, the mechanisms of lncRNA SCAMP1-involved tumorigenesis have not been reported in human osteosarcoma. In this study, we utilized multiple cellular biological approaches to determine the function of lncRNA SCAMP1 in osteosarcoma cells. Moreover, we performed several molecular biological approaches to define the mechanism by which lncRNA SCAMP1 regulated cell viability and invasion in osteosarcoma. We dissected that lncRNA SCAMP1 promoted progression of osteosarcoma *via* modulation of miR-26a-5p/ZEB2 axis. In conclusion, targeting lncRNA SCAMP1 and its downstream targets, miR-26a-5p and ZEB2, might be a useful approach for osteosarcoma therapy.

## Introduction

Osteosarcoma often occurs in children and adolescents, which affects their health due to that osteosarcoma is one of bone malignant tumors (1). Osteosarcoma patients often have a worse prognosis due to the distant metastasis, such as pulmonary metastasis (2). The conventional therapy for osteosarcoma includes surgery, neoadjuvant, radiotherapy and chemotherapy (3, 4). The five-year survival rate is unsatisfactory in osteosarcoma patients because of the lack of early detection and development of metastasis as well as radio-resistance and drug resistance (5–7). Moreover, the molecular mechanisms of osteosarcoma development and progression are not fully determined. Hence, exploration of molecular insight into osteosarcoma initiation and progression is pivotal to discover the new therapeutic strategy.

In recent years, noncoding RNAs have burst into stage in cancer development and malignant behaviors (8–12). Evidence has suggested that noncoding RNAs are involved in regulation of some cellular biological functions, such as proliferation, cell cycle, autophagy, motility, metastasis, epithelial-to-mesenchymal transition, cancer stem cell, drug resistance and immunotherapy in a variety of cancers (13–17). Noncoding RNAs conduct protein modification, epigenetic modulation, RNA degradation, chromatin remodeling, etc. (18). Based on their size, noncoding RNAs are classified into small noncoding RNAs (< 200 nucleotides), long noncoding RNAs (lncRNA, >200 nucleotides). Accumulated studies have suggested that lncRNAs are important factors to drive osteosarcoma development (19).

SCAMP1 (secretory carrier membrane protein 1) has been implied to associate with tumorigenesis. In pancreatic cancer tissues, SCAMP1 was remarkable upregulated in tissues with lymph node metastasis compared with tissues without metastasis (20). Silencing of SCAMP1 by siRNA transfection led to a marked suppression in invasion and migration in pancreatic cancer cells and gallbladder cancer cells (20). Knockdown of SCAMP1 reduced the activity of vascular endothelial growth factor (VEGF) in gallbladder cancer and pancreatic cancer (20). SCAMP1 expression was correlated with the patient clinicopathological features in pancreatic cancer, including TNM stage, neural invasion, poor prognosis (21). In breast cancer cells, SCAMP1 prevented cell invasion *via* cooperation of MTSS1 (metastasis suppressor protein 1) in breast cancer (22). Loss of SCAMP1 and MTSS1 in breast cancer tissues associated with poor disease-specific survival in HER2+ breast cancer patients (22). SCAMP1-transcript variants (SCAMP1-TV2) knockdown repressed invasion, migration and viability, and induced apoptosis in breast cancer cells (23). Depletion of SCAMP1-TV2 reduced its interaction with PUM2 and enhanced the PUM2 and INSM1 interactions, leading to INSM1 mRNA degradation in breast cancer (23).

LncRNA SCAMP1 has been discovered to play a necessary role in carcinogenesis and progression. For example, lncRNA SCAMP1 was remarkable elevated in ovarian cancer cells and tissues, and overexpression of lncRNA SCAMP1 induced angiogenesis and invasion (24). Moreover, lncRNA SCAMP1 can bind with miR-137 and upregulate the expression of CXCL12 (C-X-C motif chemokine ligand 12) in ovarian cancer cells (24). The mechanisms of lncRNA SCAMP1-mediated tumorigenesis have not been determined in human osteosarcoma. In this study, we utilized multiple cellular biological approaches to determine the function of lncRNA SCAMP1 in osteosarcoma cells. Moreover, we performed several molecular biological approaches to define the underlying mechanism by which lncRNA SCAMP1 regulated cell viability and invasion in osteosarcoma. We dissected that lncRNA SCAMP1 promoted progression of osteosarcoma *via* modulation of miR-26a-5p/ZEB2 axis.

## Materials and methods

### Cell culture

The human osteosarcoma cell lines MG63 and U2OS cells were bought from American Type Culture Collection (ATCC) Company. The MG63 and U2OS cells were maintained in DMEM medium with 1% penicillin/streptomycin and 5% fetal bovine serum at 37°C incubator under 5% CO_2_ atmosphere.

### Transfection

The human osteosarcoma MG63 and U2OS cells were seeded in 6-well plates for overnight. Then, osteosarcoma cells were transfected with negative control or miR-26a mimics, miR-26a inhibitors, ZEB2 cDNA, shR-ZEB2, shR-SCAMP1, or SCAMP1 cDNA by Lipofectamine 2000 according to the instruction’s approach as described previously (25). After different time transfection, further analysis was performed in transfected osteosarcoma cells for their viability, migration and invasion.

### Quantitative real-time reverse transcription-PCR

Total RNA was extracted from the transfected osteosarcoma cells using 1ml TRLzol Reagent. Then, 1microgram RNA was used for reverse transcription to generate first-strand cDNA. PCR was conducted using SYBR Green Kit following the manufacturer’s instructions as described before (26).

### Cell viability assay

The transfected osteosarcoma cells were seeded in 96-well plates for overnight. After 24, 48 and 72 hours, CCK8 cell viability assay was used to measure the viability of osteosarcoma cells. Briefly, after different time treatments, 10 μL CCK8 reagent was put into each well and maintained for 3 hours at 37 °C in a humidified incubator. The OD values were measured by the microplate reader at 450 nm wavelength.

### Colony formation assay

The human osteosarcoma MG63 and U2OS cells were seeded in 6-well plates and cultured with full DMEM medium for 10-14 days at 37°C incubator under 5% CO_2_ atmosphere. Then, the cells were washed by PBS three times after discarding the medium. 4% paraformaldehyde was used to fix the osteosarcoma cells for 30-40 minutes. The colonies were stained by 0.1% crystal violet for 15 minutes. The images were taken by microscope and the colony numbers were calculated.

### Transwell invasion assay

The transfected osteosarcoma cells were seeded in top Transwell plates with serum-free DMEM on 24-well plates. The bottom Transwell plates were filled with full DMEM medium, which make the cells transfer from the top plates to the bottom plates *via* the transwell membranes with Matrigel. After 20 hours, the culture medium in the top plates were removed and the plates were washed three times by PBS. Then, the plates were fixed by 4% paraformaldehyde for half hour and stained by 0.1% crystal violet. The invasive cells were imaged by an inverted microscope.

### Wound healing assay

The transfected osteosarcoma cells were seeded on 6-well plates. Until the cell fluence reached high than 90%, the pipette tip was used to scratch a wound. The cells were washed three times to remove the floatage cells. After 20 hours, the wound location was taken photographs as described before (27).

### Luciferase report assay

To confirm the interaction between lncRNA SCAMP1 and miR-26a-5p, we performed luciferase report assay. The mutant binding sequences of miR-26a-5p in lncRNA SCAMP1 were cloned into pmirGLO dual-luciferase vector. The osteosarcoma cells were transfected with various plasmids. The lncRNA SCAMP1 mutant had the mutant binding sequences of miR-26a-5p. The luciferase activity was measured following manufacturer’s instructions.

### Statistical analysis

In this study, all data were analyzed by GraphPad Prism 5.0. Student t test was performed to measure significance between two groups. ANOVE was used to compare three or more groups. The results were descripted as means ± SD. P < 0.05 was considered as statistically significant.

## Results

### LncRNA SCAMP1 overexpression stimulates viability and colony formation of osteosarcoma cells

**Figure 1:**
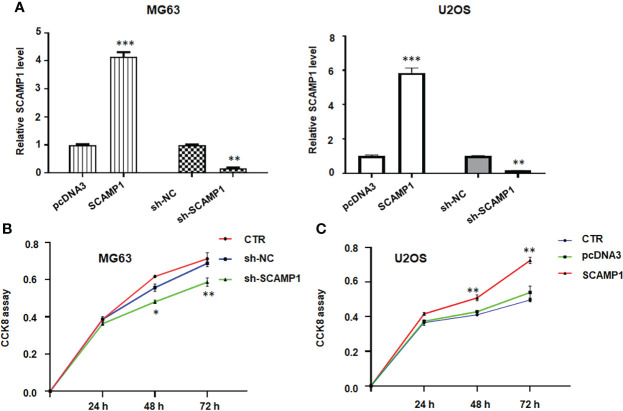
LncRNA SCAMP1 overexpression stimulates viability of osteosarcoma cells. **(A)**: RT-PCR data showed that SCAMP1 cDNA transfection increased the SCAMP1 expression, while sh-SCAMP1 transfection decreased the expression of SCAMP1 in MG63 and U2OS cells. **(B)**: CCK8 assays showed that sh-SCAMP1 transfection reduced viability of MG63 cells. **(C)**: CCK8 assays showed that SCAMP1 cDNA transfection induced viability of U2OS cells. *p<0.05; **p<0.01; ***p<0.001 *vs.* control group. CTR: control; sh-NC: shRNA negative control; sh-SCAMP1: shRNA SCAMP1; SCAMP1: SCAMP1 cDNA.

**Figure 2:**
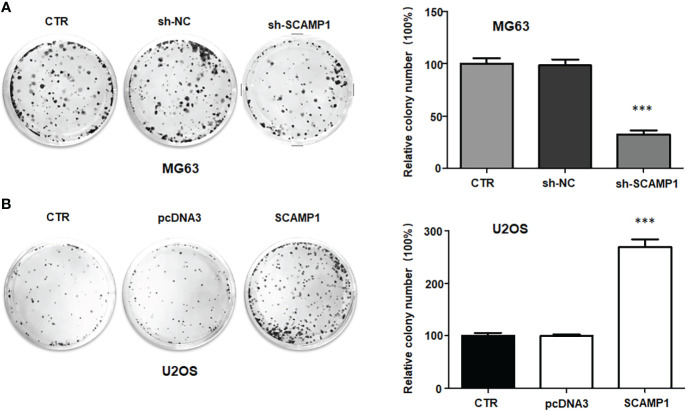
LncRNA SCAMP1 overexpression increases colony formation of osteosarcoma cells. **(A)**: Left panel: Colony formation assays showed that sh-SCAMP1 transfection reduced colony formation of MG63 cells. Right panel: quantitative data is shown for colony formation of MG63 cells. **(B)**: Left panel: Colony formation assays showed that SCAMP1 cDNA transfection increased colony formation of U2OS cells. Right panel: quantitative data is shown for colony formation of U2OS cells. ***p<0.001 *vs.* control group.

To check the function of lncRNA SCAMP1 in osteosarcoma cells, we transfected the SCAMP1 cDNA into MG63 and U2OS cells using Lipofectamine 2000. Our RT-PCR data indicated that lncRNA SCAMP1 was highly elevated in MG63 and U2OS cells after SCAMP1 cDNA transfection (**Figure 1A**). Moreover, shR-SCAMP1 was transfected into MG63 and U2OS cells by Lipofectamine 2000. We found that lncRNA SCAMP1 expression was remarkable reduced in MG63 and U2OS cells after shR-SCAMP1 transfection (**Figure 1A**). To examine whether the cell viability was governed by lncRNA SCAMP1, CCK8 assay was utilized in MG63 and U2OS cells after lncRNA SCAMP1 modulation. As expected, shR-SCAMP1 transfected MG63 cells exhibited the less viability at 48 hours and 72 hours (**Figure 1B**). Consistently, SCAMP1 cDNA transfection led to high viability at 48 hours and 72 hours in U2OS cells (**Figure 1C**). To validate this role of lncRNA SCAMP1 in osteosarcoma cells, colony formation assay was conducted in MG63 and U2OS cells after lncRNA SCAMP1 knockdown and overexpression, respectively. The data showed that knockdown of lncRNA SCAMP1 reduced the number of colony formation in MG63 cells (**Figure 2A**). In line with this result, lncRNA SCAMP1 overexpression increased the number of colony formation in U2OS cells (**Figure 2B**). Therefore, lncRNA SCAMP1 could govern the viability of osteosarcoma cells.

### LncRNA SCAMP1 overexpression increased invasion and migration of osteosarcoma cells

**Figure 3:**
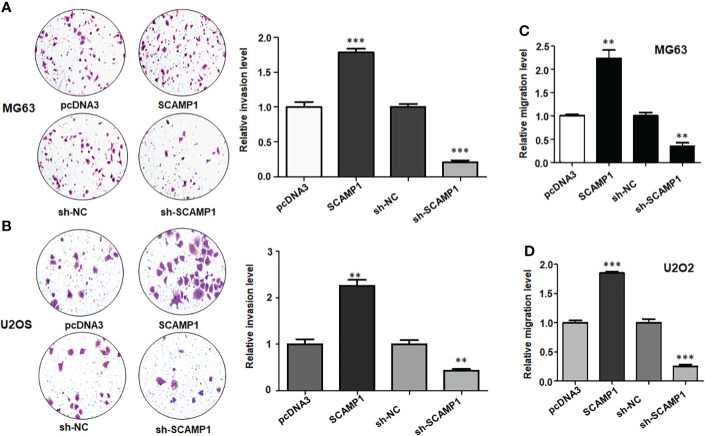
LncRNA SCAMP1 overexpression increases invasive and migratory abilities of osteosarcoma cells. **(A)**: Left panel: Transwell invasion assays showed that sh-SCAMP1 transfection reduced invasion of MG63 cells, while SCAMP1 cDNA transfection increased invasion of MG63 cells. Right panel: quantitative data is shown for invasive ability of MG63 cells. **(B)**: Left panel: Transwell invasion assays showed that sh-SCAMP1 transfection and SCAMP1 cDNA transfection modulated invasion of U2OS cells. Right panel: quantitative data is shown for invasion ability of U2OS cells. **(C)**: Wound healing assays showed that sh-SCAMP1 transfection reduced migration of MG63 cells, while SCAMP1 cDNA transfection increased migration of MG63 cells. **(D)**: Wound healing assays showed that sh-SCAMP1 transfection and SCAMP1 cDNA transfection modulated migratory ability of U2OS cells. **p<0.01; ***p<0.001 *vs.* control group.

It is known that Transwell invasion assay is a good approach for detection of cell invasive ability in cancer. We used Transwell invasion assay to test the function of lncRNA SCAMP1 in osteosarcoma cells. As shown in **Figures 3A, B**, SCAMP1 cDNA transfection resulted in promotion of invaded cell numbers in both MG63 and U2OS cells. The invasiveness ability in shR-SCAMP1 transfected osteosarcoma cells was also examined using Transwell invasion assay. Expectedly, shR-SCAMP1 transfected cells had a reduction in invaded cell numbers in U2OS and MG63 cells (**Figures 3A**, **B**). Wound healing assay is often used for measuring the migrative ability in cancer cells. Therefore, we used the wound healing assay in our study to check the role of lncRNA SCAMP1 in regulation of migration in osteosarcoma cells. The wound healing assay data showed that shR-SCAMP1 transfected cells had a slower rate to close the wound area in U2OS and MG63 cells (**Figures 3C**, **D**). On the contrary, both osteosarcoma cell lines with SCAMP1 cDNA transfection had a faster rate to close the wound area (**Figures 3C**, **D**). Hence, lncRNA SCAMP1 could govern the invasive and migrative abilities in osteosarcoma cells.

### LncRNA SCAMP1 interacts with and regulates miR-26a-5p in osteosarcoma cells

**Figure 4:**
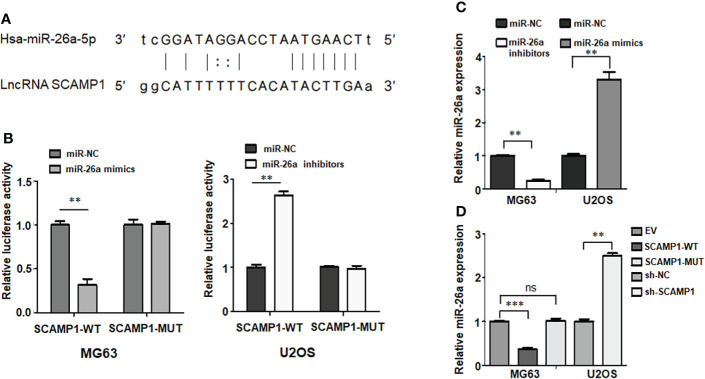
LncRNA SCAMP1 interacts with and regulates miR-26a-5p in osteosarcoma cells. **(A)**: Potential binding sites between miR-26a-5p and lncRNA SCAMP1. **(B)**: Dual luciferase reporter assays showed that miR-26a interacted with lncRNA SAMP1. miR-26a-5p modulation regulated luciferase activity in SCAMP1 wild-type group. **(C)**: RT-PCR data showed that miR-26a inhibitors decreased the expression of miR-26a-5p in MG63 cells, while miR-26a mimic transfection increased the miR-26a-5p expression levels in U2OS cells. **(D)**: RT-PCR data showed that upregulation of SCAMP1 suppressed the expression of miR-26a-5p in MG63 cells, while sh-SCAMP1 increased miR-26a-5p expression in U2OS cells. SCAMP1-WT: SCAMP1 wild type; SCAMP1-MUT: SCAMP1 mutant. **P < 0.01; ***P < 0.001 vs control group; ns, no significance.

It has been known that lncRNAs often sponge with miRNAs to suppress their expressions. Therefore, we aimed to explore the lncRNAs that could bind to lncRNA SCAMP1 in osteosarcoma cells. Based on the TargetScan database from website, we saw the binding sites between lncRNA SCAMP1 and miR-26a-5p (**Figure 4A**). The luciferase reporter gene assay is a regular approach to confirm the interaction between lncRNAs and miRNAs. The data from the dual luciferase assay showed that miR-26a-5p mimic transfection led to a reduction in the luciferase activity in the lncRNA SCAMP1 wild-type group (**Figure 4B**). On the contrary, this phenotype was not observed in the lncRNA SCAMP1 mutant group (**Figure 4B**). Moreover, miR-26a-5p inhibitor increased the luciferase activity in lncRNA SCAMP1 wild-type group, but not in lncRNA SCAMP1 mutant group (**Figure 4B**). We also measured the expression of miR-26a-5p in U2OS and MG63 cells after miR-26a-5p mimics transfection and miR-26a-5p inhibitor treatment, respectively. Our data showed that miR-26a inhibitors decreased the expression of miR-26a-5p in MG63 cells, while miR-26a mimic transfection increased the miR-26a-5p expression levels in U2OS cells (**Figure 4C**). Notably, upregulation of wild-type lncRNA SCAMP1 suppressed the expression of miR-26a-5p in MG63 cells, but this phenotype did not exhibit in mutant lncRNA SCAMP1 group (**Figure 4D**). Strikingly, shR-SCAMP1 group showed the high expression of miR-26a-5p compared with shR-NC group in U2OS cells (**Figure 4D**). Hence, lncRNA SCAMP1 can interact with and regulate miR-26a-5p inU2OS and MG63 cells.

### LncRNA SCAMP1 overexpression promotes cell viability *via* miR-26a-5p

**Figure 5:**
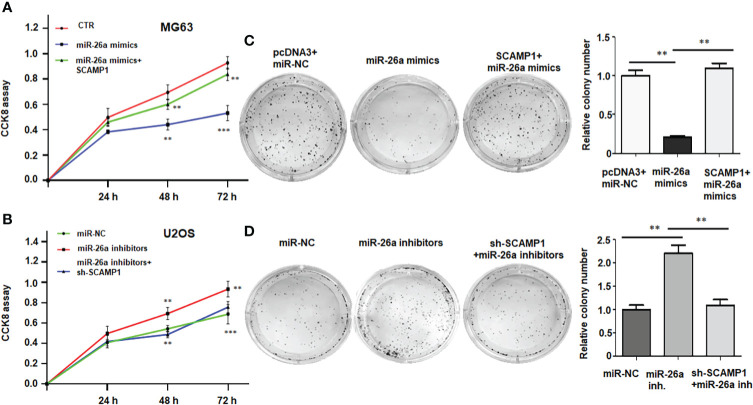
LncRNA SCAMP1 overexpression promotes cell viability and colony formation *via* miR-26a-5p. **(A)**: CCK8 assays showed that overexpression of lncRNA SCAMP1 abolished miR-26a-5p mimics-mediated inhibition of cell viability in MG63 cells. **(B)**: CCK8 assays showed that inhibition of miR-26a-5p enhanced viability of U2OS cells, which was abrogated by shR-SCAMP1 transfection. **(C)**: Left panel: Colony formation assays showed that in SCAMP1-overexpressing MG63 cells, miR-26a-5p mimics-mediated suppression of colony formation was abolished in MG63 cells. Right panel: quantitative data is shown for colony formation of MG63 cells. **(D)**: Left panel: Colony formation assays showed that in U2OS cells with shR-SCAMP1 transfection, miR-26a-5p-inhibitor-induced colony formation was blocked. Right panel: quantitative data is shown for colony formation of U2OS cells. miR-26a inh: miR-26a inhibitors. **p<0.01; ***p<0.001 *vs.* control group.

To check whether miR-26a-5p participates in lncRNA SCAMP1-mediated promotion of cell viability in osteosarcoma cells, the MG63 cells were treated with miR-26a-5p mimics in combination with lncRNA SCAMP1 cDNA transfection. Our CCK8 results indicated that miR-26a-5p mimic transfection suppressed viability of MG63 cells at 48 hours and 72 hours (**Figure 5A**). Overexpression of lncRNA SCAMP1 abolished miR-26a-5p mimics-mediated inhibition of cell viability in MG63 cells (**Figure 5A**). Moreover, inhibition of miR-26a-5p enhanced viability of U2OS cells, which was abrogated by shR-SCAMP1 transfection (**Figure 5B**). In line with these results, colony formation data revealed that miR-26a-5p mimics reduced the colony numbers in MG63 cells, while miR-26a-5p inhibitors increased the colony numbers in U2OS cells (**Figure 5C** *and* 65D). In SCAMP1-overexpressing MG63 cells, miR-26a-5p mimics-mediated suppression of colony formation was abolished in MG63 cells (**Figure 5C**). In U2OS cells with shR-SCAMP1 transfection, miR-26a-5p-inhibitor-induced colony formation was blocked in U2OS cells (**Figure 5D**). Taken together, lncRNA SCAMP1 overexpression promotes cell viability *via* miR-26a-5p in osteosarcoma cells.

### ZEB2 is a downstream target of miR-26a-5p in osteosarcoma cells

**Figure 6:**
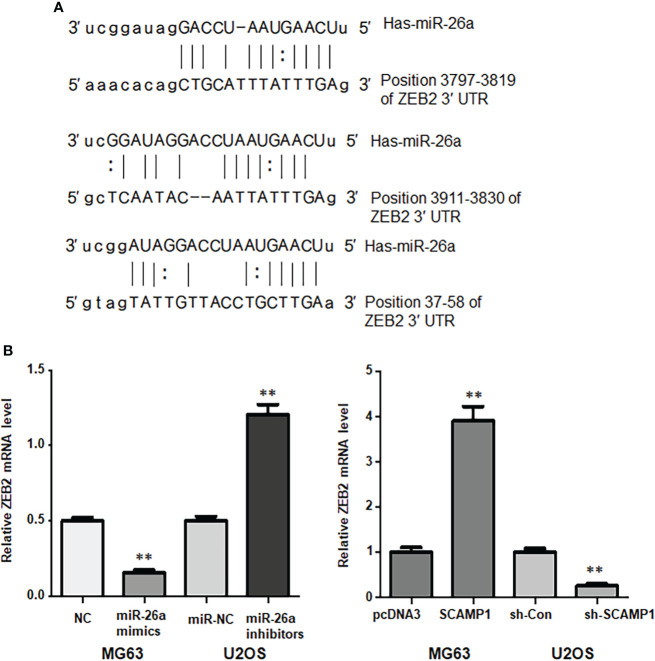
ZEB2 is a downstream target of miR-26a-5p in osteosarcoma cells. **(A)**: The potential binding sites between miR-26a-5p and ZEB2. **(B)**: Left panel: RT-PCR data showed that miR-26a-5p mimics attenuated the mRNA levels of ZEB2 in MG63 cells. miR-26a-5p inhibitors elevated ZEB2 mRNA levels in U2OS cells. Right panel: RT-PCR data showed that SCAMP1-overexpressing cells had the high expression of ZEB2 in MG63 cells. In shR-SCAMP1 transfected U2OS cells, ZEB2 mRNA levels were remarkable downregulated. **p<0.01 *vs.* control group.

**Figure 7:**
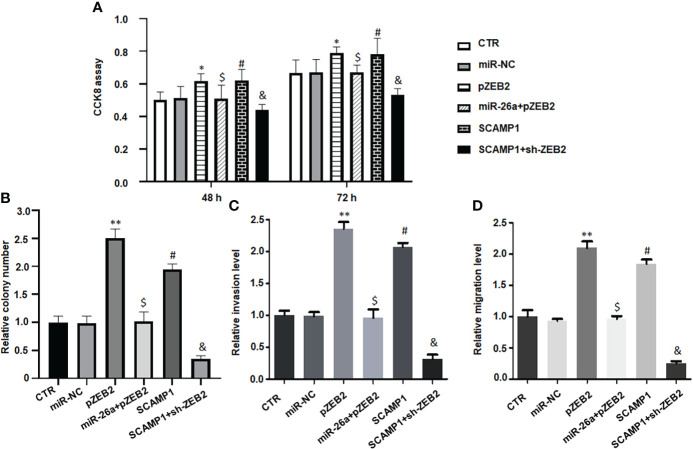
LncRNA SCAMP1 increases cell malignant progression *via* miR-26a/ZEB2 axis. **(A)**: CCK8 assays data showed that upregulation of ZEB2 abrogated miR-26a-5p-induced inhibition of cell viability in MG63 cells. SCAMP1 overexpression elevated cell viability, which was blocked by downregulation of ZEB2 in MG63 cells. **(B)**: Colony formation data showed that ZEB2 cDNA transfection increased colony formation of MG63 cells, which was abrogated by miR-26a mimics transfection. SCAMP1 upregulation elevated colony formation of MG63 cells, which was blocked by sh-ZEB2 transfection. **(C)**: Transwell invasion assay data showed that upregulation of ZEB2 abrogated miR-26a-5p-induced suppression of cell invasion in MG63 cells. SCAMP1 overexpression elevated cell invasion, which was reduced by downregulation of ZEB2 in MG63 cells. Right panel: quantitative data is shown for invasion assay of left panel. **(D)**: Wound healing assays showed that ZEB2 cDNA transfection increased migrative ability of MG63 cells, which was abrogated by miR-26a mimics transfection. LncRNA SCAMP1 upregulation elevated migrative ability of MG63 cells, which was blocked by sh-ZEB2 transfection. **p<0.01 *vs.* control group; ^$^p<0.01 *vs.* pZEB2 group; ^#^p<0.01 *vs.* control group; ^&^p<0.01 *vs.* SCAMP1 group. ; *p < 0.05 vs control group.

It is known that miRNAs performed their functions in tumorigenesis *via* inhibiting the expression of their downstream genes. According to the TargetScan database from website, we observed the binding sites between miR-26a-5p and ZEB2, indicating that miR-26a-5p could interact with ZEB2 and suppress its expression (**Figure 6A**). In MG63 cells, we found that miR-26a-5p mimics attenuated the mRNA levels of ZEB2 (**Figure 7B**). Similarly, in U2OS cells, we found that miR-26a-5p inhibitors elevated the mRNA levels of ZEB2 (**Figure 6B**). Moreover, we tested whether lncRNA SCAMP1 regulated the expression of ZEB2 in osteosarcoma cells. We found that SCAMP1-overexpressing cells had the high expression of ZEB2 in MG63 cells (**Figure 6B**). In shR-SCAMP1 transfected U2OS cells, ZEB2 mRNA levels were remarkable downregulated (**Figure 6B**). Altogether, miR-26a-5p could target ZEB2 in osteosarcoma cells.

### LncRNA SCAMP1 increases cell viability and colony formation *via* miR-26a/ZEB2 axis

To determine whether lncRNA SCAMP1 increased viability of osteosarcoma cells *via* targeting miR-26a/ZEB2 axis, MG63 cells were co-transfected with miR-26a-5p mimics and ZEB2 cDNA, or SCAMP1 and sh-ZEB2. Our CCK8 assay data demonstrated that pZEB2 cDNA transfection increased cell viability in MG63 cells (**Figure 7A**). Upregulation of ZEB2 abrogated miR-26a-5p-induced inhibition of cell viability in MG63 cells (**Figure 7A**). Moreover, SCAMP1 overexpression elevated cell viability, which was blocked by downregulation of ZEB2 in MG63 cells (**Figure 7A**). Similarly, ZEB2 cDNA transfection increased colony formation of MG63 cells, which was abrogated by miR-26a mimics transfection (**Figure 7B**). LncRNA SCAMP1 upregulation elevated colony formation of MG63 cells, which was blocked by sh-ZEB2 transfection (**Figure 7B**).

### LncRNA SCAMP1 increases cell invasion and migration *via* miR-26a/ZEB2 axis

To examine whether lncRNA SCAMP1 promoted invasion and migration of osteosarcoma cells *via* targeting miR-26a/ZEB2 axis, MG63 cells were co-transfected with miR-26a-5p mimics plus ZEB2 cDNA, or SCAMP1 plus shZEB2. Our Transwell invasion assay data dissected that pZEB2 cDNA transfection increased cell invasion ability in MG63 cells (**Figure 7C**). Upregulation of ZEB2 abrogated miR-26a-5p-induced suppression of cell invasion in MG63 cells (**Figure 7C**). Moreover, SCAMP1 overexpression elevated cell invasion, which was reduced by downregulation of ZEB2 in MG63 cells (**Figure 7C**). Similarly, ZEB2 cDNA transfection increased migrative ability of MG63 cells, which was abrogated by miR-26a mimics transfection (**Figure 7D**). LncRNA SCAMP1 upregulation elevated migrative ability of MG63 cells, which was blocked by sh-ZEB2 transfection (**Figure 7D**). In a word, lncRNA SCAMP1 regulated cell invasiveness and migrative ability through targeting miR-26a and ZEB2 pathways in osteosarcoma.

## Discussion

LncRNAs are involved in osteosarcoma development, progression, metastasis, prognosis and drug resistance (28, 29). LncRNAs utilized their functions in oncogenesis *via* targeting various cellular signaling pathways (30). For instance, lncRNA CBR3-AS1 targeted the network of miR-140-5p-DDX54-NUCKS1-mTOR signaling pathway and contributed to osteosarcoma progression (31). One study found that lncRNA DARS-AS1 regulated miR-532-3p/CCR7 axis and facilitated progression of osteosarcoma (32). Another study revealed that lncRNA SNHG1 stimulated osteosarcoma development *via* sponging miR-493-5p and elevating the expression of S100A6 (33). Moreover, lncRNA ODRUL acted as a sponge of miR-6874-3p to elevate the expression of IL-6, leading to osteosarcoma progression (34). Furthermore, lncRNA MELTF-AS1 regulated the expression of MMP14 and enhanced osteosarcoma metastasis (35). In addition, lncRNA MALAT1 sponged miR-150-5p and increased the expression of VEGFA and enhanced tumor angiogenesis in osteosarcoma (36). LncRNA PURPL affected tumor-associated macrophages through modulating miR-363 and PDZD2 in osteosarcoma cells (37). These studies indicated that lncRNAs critically participate in osteosarcoma progression.

LncRNA SCAMP1 was reported to be significantly upregulated in pancreatic cancer tissues compared with normal tissues (38). Pancreatic cancer patients with unfavorable survival often had higher expression of lncRNA SCAMP1. The mRNA-miRNA-lncRNA regulatory network predicted that lncRNA SCAMP1 could bind to miR-132-3p and MMP9 in pancreatic cancer cells (38). LncRNA SCAMP1 has been reported to regulate ZEB1/JUN axis in renal cell carcinoma (39). In renal cell carcinoma specimens, the expression of lncRNA SCAMP1 was highly elevated. Knockdown of lncRNA SCAMP1 induced apoptosis and reduced cell viability in renal cell carcinoma cells after H_2_O_2_ treatment (39). Moreover, miR-429 was found to interact with lncRNA SCAMP1 in renal cell carcinoma cells. Consistently, miR-429 expression was remarkable decreased in human renal cancer samples. Moreover, miR-429 targets both ZEB1 and JUN in renal cell carcinoma cells. Furthermore, lncRNA SCAMP1 also affected autophagy and miR-429-mediated tumorigenesis in renal cell carcinoma (39). Therefore, lncRNA SCAMP1 enhanced progression of renal cell carcinoma *via* regulation of autophagy and miR-429/ZEB1/JUN axis under oxidative stress. In our study, we reported that lncRNA SCAMP1 modulated the expression of ZEB2 *via* sponging miR-26a-5p in osteosarcoma, which led to promotion of cell viability and colony formation. It is required to investigate whether lncRNA SCAMP1 targets ZEB1 in osteosarcoma cells. Since ZEB2 is the critical driver in EMT progression, it is needed to address whether lncRNA SCAMP1 can regulate the EMT in osteosarcoma *via* targeting ZEB2.

Zong et al. reported that silencing of lncRNA SCAMP1 restrained viability, invasive and migratory abilities and induced apoptosis *via* sponging miR-499a-5p in glioma (40). LMX1A was found to be a downstream target of miR-499a-5p and participated in lncRNA SCAMP1-induced oncogenesis in glioma. LMX1A can regulate the expression of NLRC5 (NLR family, CARD domain containing 5) and activate Wnt/β-catenin signaling pathway in glioma (40). In the current study, we observed that lncRNA SCAMP1 promoted cell invasion and migration in osteosarcoma cells. Moreover, lncRNA SCAMP1 performed their functions on cell motility *via* targeting miR-26a-5p/ZEB2 axis in osteosarcoma.

## Conclusions

In summary, lncRNA SCAMP1 modulated the expression of ZEB2 *via* sponging miR-26a-5p in osteosarcoma, which resulted in promotion of viability and colony formation of osteosarcoma cells. We also reported that lncRNA SCAMP1 promoted cell invasiveness and migrative capacity in osteosarcoma cells *via* regulating miR-26a-5p/ZEB2 axis. This study has several limitations. For instance, the current study used cell culture system to define the role of lncRNA SCAMP1 in osteosarcoma. This study did not use the mouse model and clinical tissues from osteosarcoma patients to dissect the function of SCAMP1. Therefore, it is necessary to describe that *in vivo* experiments and clinical sample study are required to determine the role of lncRNA SCAMP1 in osteosarcoma.

## Data availability statement

The original contributions presented in the study are included in the article/**Supplementary Material**. Further inquiries can be directed to the corresponding author.

## Author contributions

RL and LD designed this study. RL, ZC, YZ, GM, and QY performed the experiments. YL, AdM, CZ, JR, CL, AbM, and PZ analyzed the data. RL and LD wrote the manuscript. All authors contributed to the article and approved the submitted version.

## Funding

This project is supported by the National Natural Science Foundation of China (82160449); China Postdoctoral Science Foundation (2019M663963XB); Natural Science Foundation of the Xinjiang Uygur Autonomous Region (2018D01C301); Supported by the founding from Key Laboratory of Special Environment and Health Research in Xinjiang.

## Conflict of interest

The authors declare that the research was conducted in the absence of any commercial or financial relationships that could be construed as a potential conflict of interest.

## Publisher’s note

All claims expressed in this article are solely those of the authors and do not necessarily represent those of their affiliated organizations, or those of the publisher, the editors and the reviewers. Any product that may be evaluated in this article, or claim that may be made by its manufacturer, is not guaranteed or endorsed by the publisher.
